# Transcription Factor 7-Like 2 (TCF7L2) rs7903146 Polymorphism as a Risk Factor for Gestational Diabetes Mellitus: A Meta-Analysis

**DOI:** 10.1371/journal.pone.0153044

**Published:** 2016-04-08

**Authors:** Pei-Chao Lin, Wei-Ting Lin, Yao-Hsien Yeh, Shu-Fen Wung

**Affiliations:** 1 College of Nursing, Kaohsiung Medical University, Kaoshiung, Taiwan; 2 Department of Occupational and Environmental Medicine, National Cheng Kung University Hospital, Tainan, Taiwan; 3 Department of Environmental and Occupational Health, College of Medicine, National Cheng Kung University, Tainan, Taiwan; 4 College of Nursing, The University of Arizona, Tucson, Arizona, United States of America; University of Catanzaro Magna Graecia, ITALY

## Abstract

**Background:**

There are racial and ethnic differences in the prevalence of gestational diabetes mellitus (GDM). Prior meta-analyses included small samples and very limited non-Caucasian populations. Studies to determine the relationship between transcription factor 7 like-2 (*TCF7L2)* rs7903146 polymorphism and risk of GDM in Hispanics/Latinos are recently available. The present meta-analysis was to estimate the impact of allele variants of *TCF7L2* rs7903146 polymorphism on GDM susceptibility in overall population and racial/ethnic subgroups.

**Methods:**

Literature was searched in multiple databases including PubMed, Web of Science, EMBASE (Ovid SP), Airiti Library, Medline Complete, and ProQuest up to July 2015. Allelic frequency for *TCF7L2* rs7903146 polymorphism in GDM and control subjects was extracted and statistical analysis was performed using Comprehensive Meta-Analysis (CMA) 2.0 statistical software. The association between *TCF7L2* rs7903146 polymorphism and GDM risk was assessed by pooled odd ratios (ORs) using five gene models (dominant, recessive, homozygote, heterozygote, and allele). Stratified analysis based on race/ethnicity was also conducted. The between-study heterogeneity and contribution of each single study to the final result was tested by Cochran Q test and sensitivity analyses, respectively. Publication bias was evaluated using Egger’s linear regression test.

**Results:**

A total of 16 studies involving 4,853 cases and 10,631 controls were included in this meta-analysis. Significant association between the T-allele of rs7903146 and GDM risk was observed under all genetic models, dominant model (OR = 1.44, 95% CI = 1.19–1.74), recessive model (OR = 1.35, 95% CI = 1.08–1.70), heterozygous model (OR = 1.31, 95% CI = 1.12–1.53), homozygous model (OR = 1.67, 95% CI = 1.31–2.12), and allele model (OR = 1.31, 95% CI = 1.12–1.53). Stratified analysis by race/ethnicity showed a statistically significant association between rs7903146 polymorphism and susceptibility to GDM under homozygous genetic model (TT versus CC) among whites, Hispanics/Latinos and Asians. Sensitivity analysis showed that the overall findings were robust to potentially influential decisions of the 16 studies included. No significant evidence for publication bias was observed in this meta-analysis for overall studies and subgroup studies.

**Conclusions:**

This meta-analysis showed that the T allele of *TCF7L2* rs7903146 polymorphism was associated with susceptibility of GDM in overall population in white, Hispanic/Latino and Asian sub-groups. Asians with homozygous TT allele of rs7903146 polymorphism have highest risk of GDM (OR = 2.08) followed by Hispanics/Latinos (OR = 1.80) and whites (OR = 1.51). The highest and lowest frequency of T allele of rs7903146 was found in Malaysia and South Korea, respectively. Future studies are needed to profile genetic risk for GDM among high risk Asian and Pacific Islander subgroups.

## Introduction

Gestational diabetes mellitus (GDM) is a glucose tolerance disorder leading to hyperglycemia, diagnosed for the first time in pregnancy [1]. GDM is a global public health concern and its prevalence is increasing yearly. The prevalence of GDM ranges from 1.8% to 25.1% of all pregnancies, depending on the population studied and diagnostic tests used [2]. GDM has short- and long-term adverse outcomes both in women and their offspring, resulting in an increase in medical costs [3]. Women with a history of GDM are at over seven-fold higher risk of developing type 2 diabetes mellitus (T2DM) later in life than those without [4], however data on the risk of progression from GDM to T2DM are still limited [2]. Although the exact pathophysiology of GDM is still unclear, it is generally believed that GDM and T2DM share the same underlying pathologic mechanisms, including insulin resistance and β-cell dysfunction leading to metabolic changes [5]. In addition, T2DM is a multifactorial disease and GDM may share genetic risk factors with T2DM [6].

There are racial and ethnic differences in the prevalence of GDM. As reported by some studies [7, 8], Asian/Pacific Islander women have a higher prevalence of GDM than non-Hispanic white, Black, or Hispanic women. A contemporary estimate of global prevalence of GDM shows great regional and social economic variations [2]. Developing and low-to-middle income countries suffer from escalating burden of GDM and T2DM [2]. The prevalence of GDM is higher among Middle East and North Africa, South Asia, and Western Pacific regions and the prevalence is lowest in Europe [2]. Because of racial and regional differences of GDM prevalence, several studies have focused on exploring relationship of susceptible T2DM genes in women with GDM of different racial background [9–13].

Among common genetic variants associated with T2DM identified thus far, single nucleotide polymorphism rs7903146 of the transcription factor 7 like-2 (*TCF7L2*) gene produces the strongest susceptibility for T2DM [14,15] and this relationship is reproducibly shown in various ethnic groups [16]. *TCF7L2* is a commonly investigated gene in women with GDM [6]. It is located at chromosome 10q25.3 and its product is a high mobility group (HMG) box-containing transcription factor that is implicated in blood glucose homeostasis [17] in the morphogenic wingless-type MMTV integration site family (Wnt) signaling pathway [18]. This pathway plays an essential role in regulation of pancreatic β-cell proliferation and synthesis of incretin hormones, glucagon-like peptide 1 (GLP-1) and glucose-dependent insulinotropic peptide (GIP), in the enteroendocrine cells [19, 20]. Evidence suggests that TCF7L2 is a major regulator of insulin production and processing in pancreatic islet [21]. TCF7L2 plays a central role in coordinating the expression and subsequent processing of proinsulin to form mature insulin via several TCF7L2-target genes and the downstream regulatory network [21]. In addition, TCF7L2 may also influence hepatic clearance of insulin [21] as well as peripheral or whole body insulin sensitivity [22, 23].

The risk T allele of rs7903146 in the *TCF7L2* gene is strongly associated with an increased risk of T2DM and this effect is additive. Approximately 10% of population has two copies of the risk T allele and these individuals are twice as likely to develop T2DM as compared with individuals with no risk alleles [24]. A meta-analysis of 27 different studies confirms the association of the *TCF7L2* rs7903146 risk T allele with T2DM with a resulting global odd ratio (OR) of 1.46 [1.42–1.51] [16]. Such reproducible results among studies is indicative of a universal contribution of this gene to T2DM, thus, the population-attributable risk of diabetes is driven by the prevalence of the at risk T allele in a specific ethnic group [16]. The exact mechanisms of *TCF7L2* in the development of diabetes have not been fully determined but it is suggested that diabetes arises as a consequence of reduced pancreatic islet mass and/or impaired function [25]. The risk T allele is associated with impaired insulin secretion and incretin effects as well as enhanced rate of hepatic glucose production [17, 21]. In a longitudinal cohort with up to 22 years of follow-up, carriers of the risk T-allele had a lower insulin response to an initial oral glucose tolerance test (OGTT) and a higher risk of future T2DM than those with CC homozygotes [17]. In addition, among patients converted to T2DM after the initial screening, those carrying the risk T-allele had more severe progressive deterioration in insulin secretion as compared to those with the CC genotype [17]. The rs7903146 in *TCF7L2* gene is shown to be associated with GDM in different populations, including Korean, European Caucasian, and Mexican-American [9,26–29]. As reported in two meta-analyses, the risk variants in the *TCF7L2* gene increase risk of GDM with an effect size similar to that reported in T2DM [13, 30]. These prior meta-analyses included small samples of women with GDM and very limited non-Caucasian populations. The lack of racial/ethnic diversity in these two meta-analyses has limited the investigation of association between *TCF7L2* gene rs7903146 polymorphism and GDM in racial/ethnic subgroups. In addition, studies to determine the relationship between *TCF7L2* rs7903146 polymorphism and risk of GDM in Hispanics/Latinos were not available until recently [29, 31, 32]. Understanding genetic contributions to GDM may help to identify targets for pharmacological and non-pharmacological personalized prevention and treatment strategies.

## Materials and Methods

### Search Strategy

A broad search was performed for reports on *TCF7L2* rs7903146 polymorphism and GDM in PubMed, Web of Science, EMBASE (Ovid SP), Airiti Library, Medline Complete, and ProQuest. The keywords used for searching were “gestational diabetes” in combination with “*TCF7L2*” and the search was not limited to English language. All articles published up to June of 2015 were included. To further identify eligible studies, reference lists from the retrieved articles were also examined.

### Inclusion and exclusion criteria

Published studies meeting the following criteria were selected: (1) study subjects must be humans; (2) study design conformed to case-control; (3) study was published as an original article or a conference abstract with original data including allelic frequencies and their distributions in cases and controls; and (4) study investigated the relationship between *TCF7L2* gene and GDM. If an article did not include information on genotypic frequencies in cases and controls, the researcher contacted the study authors by mail to request specific additional data. Studies were excluded if they met any of the following criteria: (1) overlapping and insufficient data; (2) family-based studies or case only design; (3) review articles; (4) rs7903146 polymorphism was not investigated.

### Data extraction

The studies were reviewed by two independent reviewers who extracted the following information: name of the first author, year of publication, country of study setting, race/ethnicity of study subjects, sample size, mean age of cases and controls, allelic frequencies and their distributions in cases and controls, adjusted OR and 95% confidence intervals (CI), and p value for Hardy-Weinberg Equilibrium (HWE) test. Disagreements concerning inclusion/exclusion of studies or risk estimates were resolved by consensus.

### Statistical analysis

The meta-analysis was performed using Comprehensive Meta-Analysis (CMA) 2.0 statistical software (Biostat Inc., Englewood, New Jersey, USA). The strength of association between rs7903146 polymorphism and GDM risk was assessed by calculating the logarithm of OR with 95% CI. We calculated the OR by genotype and allele model comparisons of rs7903146 polymorphism between cases and controls. Stratified analysis was performed for race/ethnicity. I2 statistical test was performed to calculate the degree of inconsistency. I2 is the ratio of true heterogeneity to total variation in observed effects, representing a signal to noise ratio [33]. I2 values were calculated and used to quantify the percentages of total variation across studies that were due to heterogeneity rather than chance, with I2 > 25%, 50%, and 75% indicating low, moderate, and high heterogeneity, respectively [34]. I2 is preferable to a test for heterogeneity in judging consistency of evidence and its use is not inherently dependent on the number of studies in the meta-analysis [34]. When moderate or high between-study variation was found, a random- effect model was used to estimate the pooled ORs with their corresponding 95% CIs [35]. Two-sided *p* values less than 0.05 were considered statistically significant [36].

HWE deviation was assessed in controls of each study by chi-square test. Publication bias was examined in funnel plots and with the Egger’s regression test [37] and a p value of < 0.1 was considered statistically significant for asymmetry [37,38]. Sensitivity analysis based on the leave-one-out method was performed to evaluate the influence of each individual study on the overall results [39]. Furthermore, JMP Statistical Discovery software (Version 12 SAS institute Inc., Cary, NC) was used to create global maps to demonstrate variations in the geographical distributions of the risk allele frequency.

## Results

### Literature selection and characteristics of eligible studies

**Fig 1:**
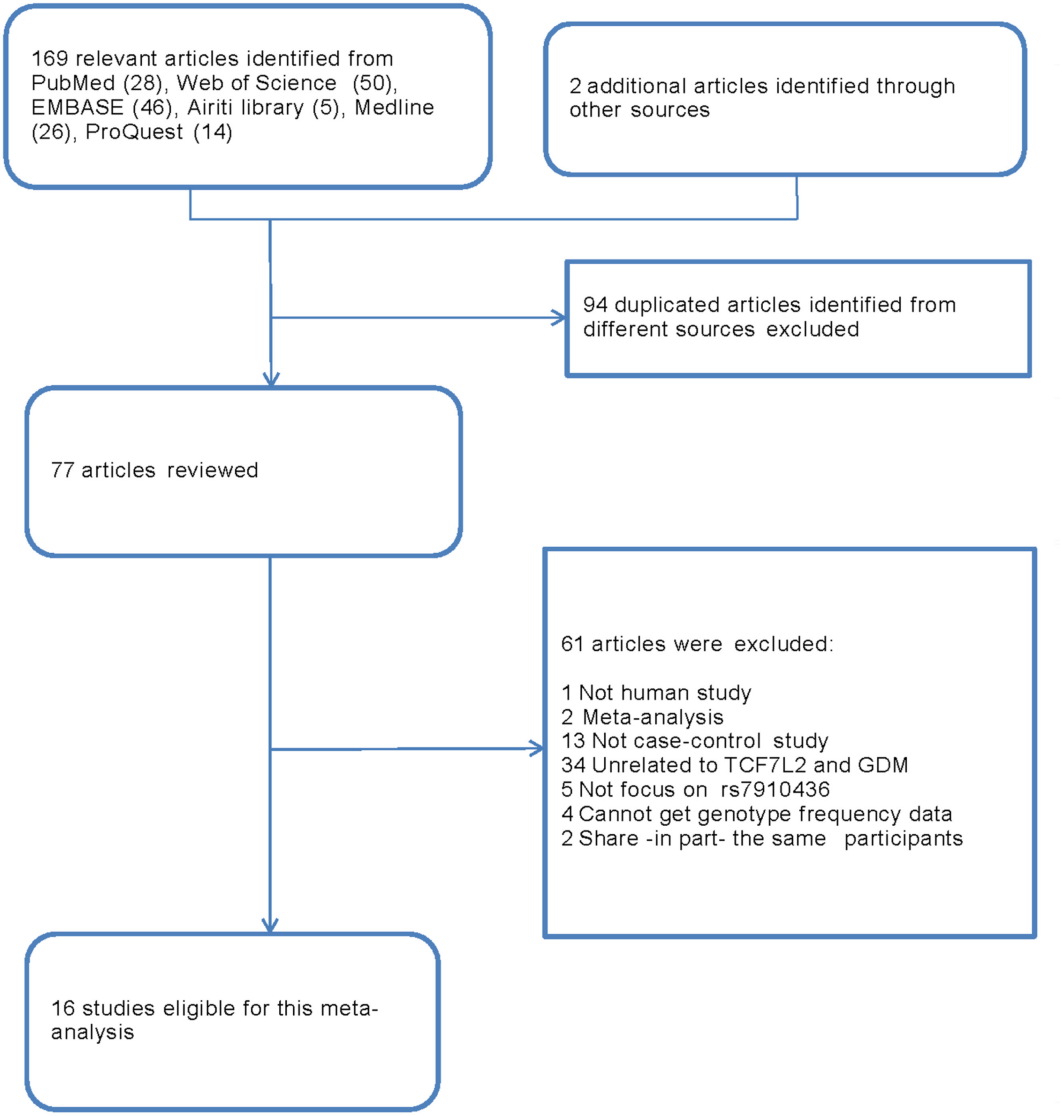
Selection of studies for inclusion in meta-analysis.

Our meta-analysis was performed according to the “Preferred Reporting Items for Systematic Reviews and Meta-analyses” (PRISMA) (S1 Table) and the “Meta-analysis on Genetic Association Studies” (S2 Table). Literature search and selection process flow chart is shown in Fig 1. One hundred and seventy-one articles were initially identified through literature search. These included 28 from PubMed, 50 from Web of Science, 46 from EMBASE (Ovid SP), 5 from Airiti Library, 26 from Medline Complete, 14 from ProQuest, and one article identified through google website search [40] and another article identified through reference list of a meta-analysis [41]. Same articles (n = 94) identified from different databases were initially excluded. Subsequently, additional 61 articles were excluded for the following reasons: (1) one article on non-human model of GDM [42]; (2) two meta-analyses on association between *TCF7L2* polymorphism and GDM; (3) 13 non case-control studies; (4) 34 studies unrelated to *TCF7L2* gene and GDM; (5) five studies not focus on rs7903146 polymorphism; (6) four articles lacking genotype frequency data in the text or from corresponding authors [43–46]; (7) two abstracts [47–48] sharing -in part- the same participants with other articles [46, 49]. Hence, 16 studies published between 2007 and 2015 were ultimately retained in this meta-analysis [9, 10, 26–29, 31, 32, 40, 41, 49–54] (S1 Text).

Characteristics of 4,853 GDM cases and 10,631 controls included in this meta-analysis are displayed in Table 1. Study countries, racial/ethnic categories, sample sizes, as well as genotype frequencies of *TCF7L2* rs7903146 polymorphism are detailed in Table 2.

**Table 1:** Characteristics of 4,853 GDM cases and 10,631 controls included in this meta-analysis.

Author	Year	GDM Mean age ± SD	Control Mean age ± SD	Controls source	GDM criteria
Aris	2012	29.7 ± 4.7	28.5 ± 3.6	Pregnant women with NGT	ADA [55]
Cho	2009	32.0 ± 3.9	Women 64.4 ± 3.3 Men 64.9 ± 3.8	Age ≥ 60 years, no history of T2DM, no first-degree relatives with T2DM, fasting plasma glucose level < 6.1 mmol/L and HbA1C level < 5.8%	NDDG [56]
de Melo	2015	33.0 ± 6.4	24.9 ± 4.0	Pregnant women with NGT	ADA [57]
Freathy	2010	NA	NA	Pregnant women with NGT	IADPSG [58]
Huerta-Chagoya	2015	28	35	Pregnant women with NGT	Carpenter and Coustan (1982) [59]
Klein	2012	30.1 ± 3.4	28.2 ± 4.8	Pregnant women with NGT	IADPSG [58]
Lauenborg	2009	43.1	45.2	Middle-aged women with NGT	50-g OGTT (year 1978–1985)[60], 75-g OGTT (year 1987–1996) [61]
Pagán	2014	31.2 ± 0.95	34.31 ± 0.63	Pregnant women with NGT	NDDG [56]
Papadppoulou	2011	31 (Median)	30 (Median)	Pregnant women with NGT	Lind et al. [58]
Pappa	2011	32.5 ± 4.5	26.67 ± 3.87	Pregnant women with NGT	ADA [62]
Reyes-López	2014	31 ± 7	29 ± 8	Pregnant women with NGT	ADA [57]
Rizk	2011	NA	NA	NA	NA
Shaat	2007	32.3 ± 0.2	30.5 ± 0.1	Pregnant women with NGT	Lind et al. (1991) [58]
Shi	2014	30 ± 5	29 ± 4	Pregnant women with NGT	IADPSG [63]
Thomas	2014	NA	NA	Pregnant women with NGT	NA
Vcelak	2012	32.8 ± 4.9	Women 29.9 ± 10.8 Men 29.4 ± 7.8	Healthy controls without family history of T2DM, PCOS, and GDM	NA

ADA, American Diabetes Association; GDM, gestation diabetes mellitus; HbA1C, hemoglobin A1C; IADPSG, International Association of Diabetes and Pregnancy Study Groups; OGTT, oral glucose tolerance test; NDDG, National Diabetes Data Group; NGT, normal glucose tolerance; PCOS, polycystic ovary syndrome; T2DM, type 2 diabetes mellitus

**Table 2:** Study countries, racial/ethnic categories, sample sizes, and genotype frequencies of TCF7L2 rs7903146 polymorphism of studies included.

Author	Year	Country	Race/Ethnicity	GDM (n)	Control (n)	GDMCC	Control CC	GDMCT	ControlCT	GDMTT	ControlTT	p for HWE test
Aris	2012	Malaysia	Asian	173	114	1	0	43	15	129	99	0.452
Cho	2009	South Korea	Asian	868	627	803	596	63	31	2	0	0.526
de Melo	2015	Brazil	Hispanic/Latino	200	200	76	98	104	86	20	16	0.633
Freathy	2010	United Kingdom	White	614	3811	293	1884	246	1557	75	370	0.066
Huerta-Chagoya	2015	Mexico	Hispanic/Latino	408	342	265	265	124	67	19	10	0.030*
Klein	2012	Australia	White	125	125	10	11	110	106	5	8	0.000*
Lauenborg	2009	Denmark	White	276	2353	118	1292	125	863	33	198	0.002*
Pagán	2014	Spain	White	45	24	19	10	18	12	8	2	0.540
Papadppoulou	2011	Sweden	White	803	1110	363	644	352	384	88	82	0.020*
Pappa	2011	Greece	White	148	107	49	62	81	38	18	7	0.720
Reyes-López	2014	Mexico	Hispanic/Latino	90	108	55	81	29	23	6	4	0.165
Rizk	2011	Qatar	White	40	74	16	29	18	37	6	8	0.451
Shaat	2007	Sweden	White	585	1111	271	650	255	392	59	69	0.339
Shi	2014	China	Asian	100	100	40	55	36	38	24	7	0.901
Thomas	2014	India	Asian	117	49	55	27	46	18	16	4	0.686
Vcelak	2012	Czech Republic	White	261	376	142	156	102	185	17	35	0.058

* p value < 0.05

GDM, gestational diabetes mellitus; HWE, Hardy-Weinberg Equilibrium

### Association between TCF7L2 rs7903146 polymorphism and GDM risk

Association between *TCF7L2* rs7903146 polymorphism and GDM risk is shown in Table 3. Significant associations between the at risk T-allele of rs7903146 and GDM risk were observed under all gene models: the dominant model (TT + CT versus CC; OR = 1.44, 95% CI = 1.19–1.74, *p* < 0.001), the recessive model (TT versus CT + CC: OR = 1.37, 95% CI = 1.19–1.57, *p* < 0.001), the heterozygote model (TT versus CT: OR = 1.17, 95% CI = 1.01–1.35, *p* = 0.035), the homozygous model (TT versus CC: OR = 1.63, 95% CI = 1.31–1.89, *p* < 0.001), and the allele model (T-allele versus C-allele: OR = 1.31, 95% CI = 1.12–1.53, *p* = 0.001).

**Table 3:** Association between TCF7L2 rs7903146 polymorphisms and risk of gestational diabetes mellitus in overall sample and in sub-racial groups.

Genotype (number of studies)	Type of Model	Test of Heterogeneity	Statistical Model	Test of Association		Test of Publication Bias
		*I*^*2*^ (%)		Odd Ratio (95% Cl)	*p*	Egger’s *p*
TT+ CT vs. CC (16)	Dominant	74.5	Random	1.44 (1.19–1.74)	< 0.001	0.938
TT vs. CC+CT (16)	Recessive	48.7	Fixed	1.37 (1.19–1.57)	< 0.001	0.851
TT vs. CT (16)	Heterozygous	17.7	Fixed	1.17 (1.01–1.35)	0.035	0.868
TT vs. CC (16)	Homozygous	45.3	Fixed	1.63 (1.31–1.89)	< 0.001	0.781
T vs. C allele (16)	Allele	56.5	Random	1.31 (1.12–1.53)	0.001	0.952
**Subgroups**						
**White**						
TT+ CT vs. CC (9)	Dominant	84.7	Random	1.31 (1.00–1.71)	0.053	0.801
TT vs. CC+CT (9)	Recessive	22.8	Fixed	1.39 (1.19–1.62)	< 0.001	0.684
TT vs. CT (9)	Heterozygous	0	Fixed	1.20 (1.03–1.41)	0.022	0.763
TT vs. CC (9)	Homozygous	61.6	Random	1.51 (1.12–2.05)	0.007	0.764
T vs. C allele (9)	Allele	66.5	Random	1.24 (1.02–1.51)	0.028	0.729
**Hispanic/Latino**						
TT+ CT vs. CC (3)	Dominant	0	Fixed	1.76 (1.40–2.22)	< 0.001	0.960
TT vs. CC+CT (3)	Recessive	0	Fixed	1.47 (0.91–2.38)	0.114	0.420
TT vs. CT (3)	Heterozygous	0	Fixed	1.05 (0.64–1.74)	0.848	0.185
TT vs. CC (3)	Homozygous	0	Fixed	1.80 (1.01–2.94)	0.020	0.377
T vs. C allele (3)	Allele	0	Fixed	1.55 (1.19–2.03)	0.001	0.775
**Asian**						
TT+ CT vs. CC (4)	Dominant	0	Fixed	1.58 (1.16–2.14)	0.004	0.264
TT vs. CC+CT (4)	Recessive	82.9	Random	1.62 (0.43–6.13)	0.480	0.480
TT vs. CT (4)	Heterozygous	77.8	Random	1.41 (0.41–4.79)	0.585	0.456
TT vs. CC (4)	Homozygous	0	Fixed	3.08 (1.53–6.18)	0.002	0.397
T vs. C allele (4)	Allele	62.0	Random	1.28 (0.73–2.25)	0.384	0.077

dominant model: TT + CT versus CC; recessive model: TT versus CC+CT; heterozygote model: TT versus CT; homozygous model: TT versus CC; allele model: T allele versus C allele. Note: When I^2^ was greater than 50%, indicating moderate or high between-study variations, a random-effect statistical model was used to estimate the pooled odds ratios with their corresponding 95% confidence intervals (CIs).

**Fig 2:**
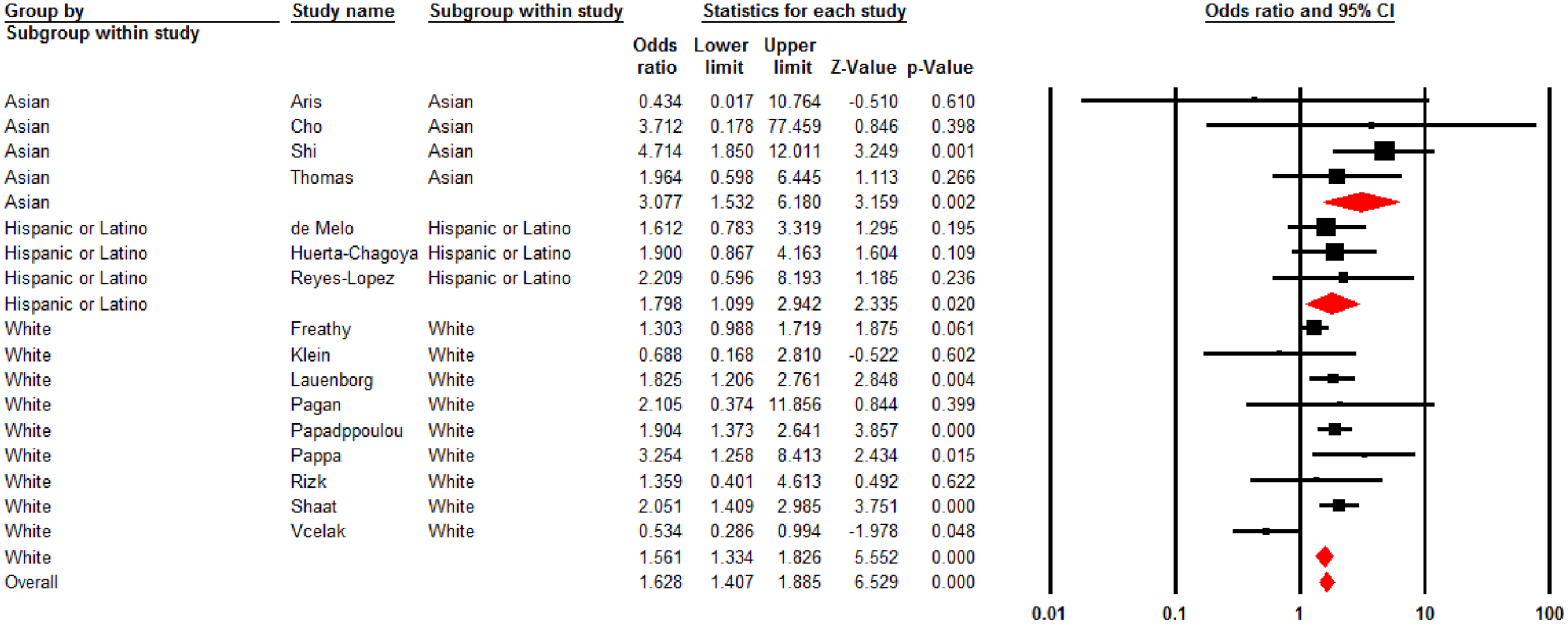
Forest plot of TCF7L2 rs7903146 polymorphism (TT versus CC) and GDM risk under fixed effect model in overall sample and sub-racial groups. The squares and horizontal lines correspond to the study specific odds ratios (ORs) and 95% confidence intervals (CI) respectively. The diamond represents the pooled ORs and 95% CI.

Stratified analysis by race/ethnicity showed significant association between rs7903146 polymorphism and GDM risk under homozygous models (TT versus CC) in whites (OR = 1.51, 95% CI = 1.12–2.05, *p* = 0.007), Hispanics/Latinos (OR = 1.80, 95% CI = 1.01–2.94, *p* = 0.020) and Asians (OR = 2.08, 95% CI = 1.53–6.18, *p* = 0.002) (Table 3). Under allele model, presence of T allele as compared to the C allele had increased GDM risk in whites and Hispanics/Latinos but not in Asians. Under recessive and heterozygote models, significant associations between rs7903146 polymorphism and GDM risk were only observed in whites. Fig 2 depicts the forest plot summarizing the effect of T-allele versus C-allele of the rs7903146 polymorphism and risk of GDM under fixed effect model in overall sample and sub-racial groups.

**Fig 3:**
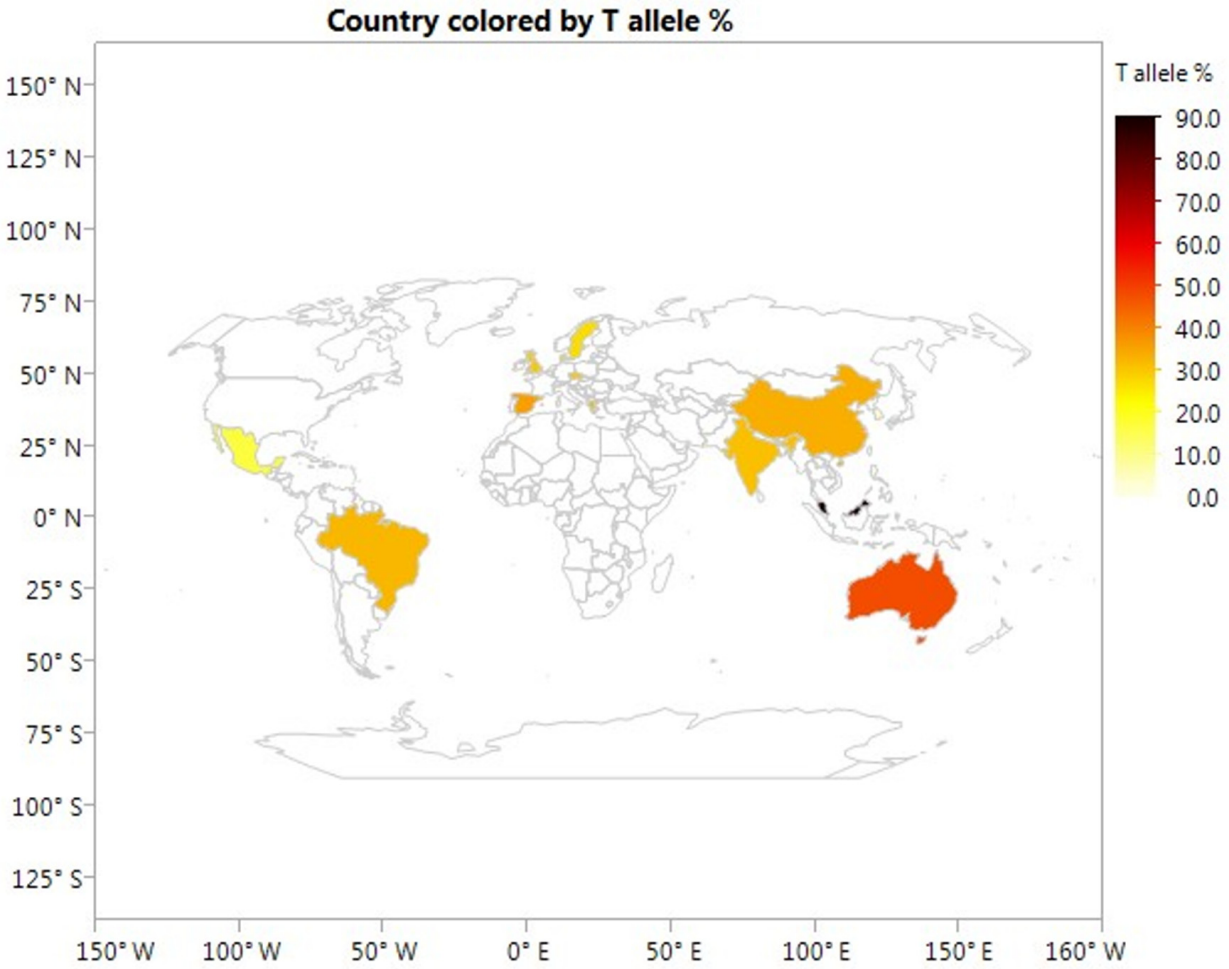
Geographic distribution of risk T allele of rs7903146 polymorphism stratified by study country. The color bar on the right corresponds to the respective allele frequency map (10% interval). This global map showing geographical distributions of the risk T allele frequency was generated using JMP Statistical Discovery software (Version 12 SAS institute Inc., Cary, NC).

**Fig 4:**
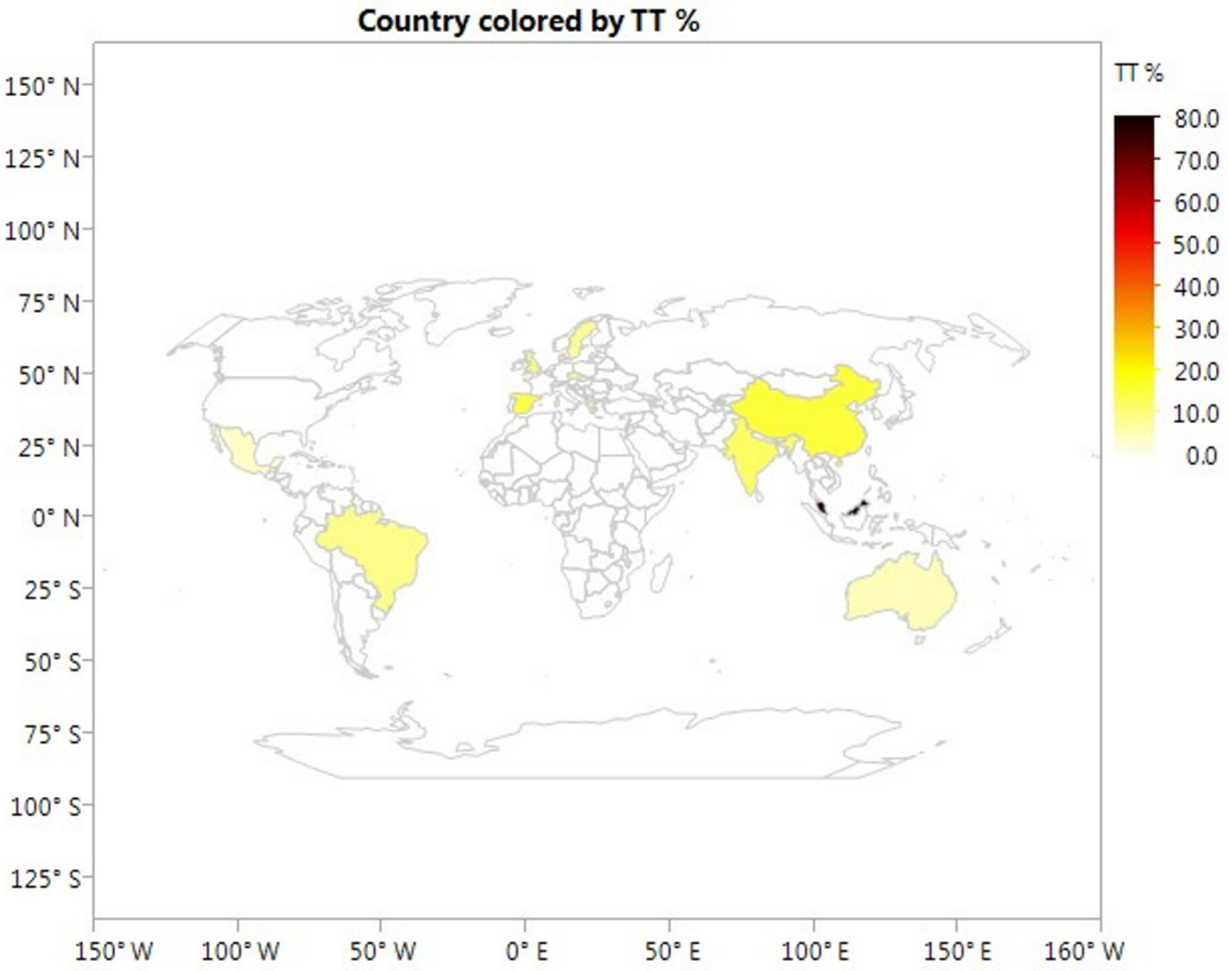
Geographic distribution of risk TT allele of rs7903146 polymorphism stratified by study country. The color bar on the right corresponds to the respective allele frequency map (10% interval). This global map showing geographical distributions of the risk T allele frequency was generated using JMP Statistical Discovery software (Version 12 SAS institute Inc., Cary, NC).

Allele frequencies of rs7903146 polymorphism by study countries are showed in Table 4. The highest frequency of T allele was found in Malaysia (89.5%) and the lowest frequency of T allele was found in South Korea (3.3%). Geographic distributions of risk T and TT alleles of rs7903146 polymorphism among subjects included in this meta-analysis stratified by study country are presented in Figs 3 and 4, respectively.

**Table 4:** The allele frequencies of rs7903146 polymorphism by country.

Country	Author	Race/Ethnicity	Total (n)	CC (%)	CT (%)	TT (%)	C allele (%)	TT allele (%)
Australia	Klein	White	250	8.4	86.4	5.2	51.6	48.4
Brazil	de Melo	Hispanic/ Latino	400	43.5	47.5	9.0	67.3	32.8
China	Shi	Asian	200	47.5	37.0	15.5	66.0	34.0
Czech Republic	Vcelak	White	637	46.8	45.0	8.2	69.3	30.7
Denmark	Lauenborg	White	2629	53.6	37.6	8.8	72.4	27.6
Greece	Pappa	White	255	43.5	46.7	9.8	66.9	33.1
India	Thomas	Asian	166	49.4	38.6	12.0	68.7	31.3
South Korea	Cho	Asian	1495	93.6	6.3	0.1	96.7	3.3
Malaysia	Aris	Asian	287	0.4	20.2	79.4	10.5	89.5
Mexico	Reyes-Lopez & Huerta-Chagoya	Hispanic/Latino	948	70.3	25.6	4.1	83.1	16.9
Qatar	Rizk	White	114	39.5	48.2	12.3	63.6	36.4
Spain	Pagan	White	69	42.0	43.5	14.5	63.8	36.2
Sweden	Papadppoulou & Shaat	White	3609	53.4	38.3	8.3	72.6	27.4
United Kingdom	Freathy	White	4425	49.2	40.7	10.1	69.6	30.4

### Sensitivity analysis

To evaluate the robustness of the association results, leave-one-out sensitivity analysis was conducted by removing one study at a time and recalculating the summary ORs. The summary ORs remained stable (Table 5), indicating that our results were not driven by any single study.

**Table 5:** Leave-one-out sensitivity analyses: Meta-analysis fixed-effects estimates when a study is omitted at a time.

Study Excluded	TT+CT vs. CC			TT vs. CC+CT			TT vs. CT			TT vs. CC			T allele vs. C allele		
	p	OR	95% CI	p	OR	95% CI	p	OR	95% CI	p	OR	95% CI	p	OR	95% CI
Aris	< 0.001	1.44	1.19–1.74	< 0.001	1.45	1.25–1.73	0.006	1.23	1.06–1.43	< 0.001	1.68	1.31–2.15	< 0.001	1.34	1.16–1.55
Cho	0.001	1.43	1.17–1.74	0.012	1.35	1.07–1.70	0.037	1.17	1.01–1.35	< 0.001	1.66	1.30–2.12	0.002	1.30	1.10–1.52
de Melo	0.001	1.43	1.16–1.74	0.014	1.36	1.06–1.74	0.032	1.18	1.01–1.36	< 0.001	1.67	1.29–2.17	0.002	1.31	1.11–1.54
Freathy	< 0.001	1.48	1.22–1.80	0.024	1.37	1.04–1.79	0.153	1.13	0.96–1.34	< 0.001	1.74	1.33–2.28	0.001	1.34	1.13–1.58
Huerta-Chagoya	0.001	1.40	1.15–1.71	0.018	1.34	1.05–1.70	0.033	1.17	1.01–1.36	< 0.001	1.65	1.28–2.14	0.003	1.28	1.09–1.51
Klein	< 0.001	1.45	1.19–1.76	0.005	1.30	1.10–1.75	0.025	1.18	1.02–1.37	< 0.001	1.71	1.34–2.18	< 0.001	1.33	1.13–1.57
Lauenborg	0.001	1.42	1.15–1.74	0.026	1.34	1.04–1.73	0.045	1.17	1.00–1.37	< 0.001	1.65	1.26–2.17	0.003	1.29	1.09–1.54
Pagán	< 0.001	1.45	1.19–1.76	0.014	1.34	1.06–1.69	0.043	1.16	1.01–1.34	< 0.001	1.66	1.29–2.13	0.001	1.31	1.12–1.54
Papadppoulou	0.001	1.41	1.14–1.74	0.032	1.33	1.03–1.73	0.057	1.17	1.00–1.37	0.001	1.64	1.24–2.17	0.004	1.29	1.08–1.53
Pappa	0.001	1.38	1.14–1.66	0.019	1.33	1.05–1.68	0.038	1.17	1.01–1.35	< 0.001	1.61	1.26–2.06	0.002	1.28	1.09–1.50
Reyes-López	0.001	1.41	1.16–1.72	0.015	1.34	1.06–1.70	0.036	1.17	1.01–1.35	< 0.001	1.65	1.29–2.13	0.002	1.29	1.10–1.52
Rizk	< 0.001	1.46	1.20–1.77	0.014	1.35	1.06–1.71	0.041	1.16	1.01–1.35	< 0.001	1.68	1.31–2.16	0.001	1.32	1.12–1.55
Shaat	0.001	1.42	1.15–1.75	0.034	1.32	1.02–1.69	0.087	1.15	0.98–1.34	< 0.001	1.62	1.24–2.12	0.004	1.29	1.08–1.53
Shi	0.001	1.41	1.16–1.72	0.019	1.29	1.04–1.59	0.082	1.14	0.98–1.32	< 0.001	1.58	1.26–1.98	0.002	1.28	1.09–1.50
Thomas	< 0.001	1.44	1.18–1.75	0.016	1.34	1.06–1.70	0.041	1.16	1.01–1.35	< 0.001	1.66	1.29–2.14	0.001	1.31	1.11–1.53
Vcelak	< 0.001	1.55	1.34–1.80	0.001	1.43	1.15–1.78	0.023	1.19	1.02–1.38	< 0.001	1.75	1.50–2.05	< 0.001	1.38	1.22–1.57

CI, confidence interval; OR, odds ratio

### Publication bias analysis

Publication bias was determined using Egger’s test. No statistically significant evidence of publication bias was observed for studies included in the overall and subgroup analyses (all p values for Egger’s test were > 0.1) (Table 3).

## Discussion

The result of this meta-analysis indicates that the at risk T allele of *TCF7L2* rs7903146 polymorphism was significantly associated with the risk of GDM in overall sample as well as in racial/ethnic subgroups, Whites, Hispanics/Latinos and Asians. The T allele of *TCF7L2* rs7903146 polymorphism was associated with a reduced compensation of insulin secretion for insulin resistance induced by 9 days of bed rest [64].

Mao and colleagues [13] performed a meta-analysis to determine the relationship between multiple polymorphisms in seven genes and GDM. Of the six studies included (four studies on Caucasians and two on East Asians), they reported that the risk T allele of TCFL2 rs7903146 polymorphism is associated with GDM for East Asians and Caucasians. Subsequently, Kang and associates [30] included 10 studies (3,404 cases and 6,473 controls) in a meta-analysis and showed a significant association between *TCF7L2* rs7903146 polymorphism and risk of GDM in the dominant and co-dominant models (ORs of 1.653 and 1.525, respectively). Of the 10 studies considered, all included Caucasians except for three studies (one study from South Korea, one from Qatar, and another from Malaysia). Due to small sample sizes with limited racial/ethnic subgroups, evaluating the relationship between *TCF7L2* rs7903146 polymorphism and the risk of GDM among racial/ethnic subgroups was limited. Our meta-analysis included an extensive dataset from 16 distinct studies with 4,853 GDM cases and 10,631 controls and showed a significant association between *TCF7L2* rs7903146 polymorphism and risk of GDM. The odd ratios in our meta- analysis are similar to the two existing meta-analyses when data are pooled disregard racial/ethnic subgroups. However, studies to determine the relationship between *TCF7L2* rs7903146 polymorphism and risk of GDM in Hispanics/Latinos were not available until recently [29, 31, 32]. In our meta-analysis, we were not only able to incorporate these recent Hispanics/Latinos studies but also one study from China [53] and another from India [40] to perform analyses in racial/ethnic subgroups.

Of racial/ethnic groups in the United States, Asian and Pacific Islander women were found to have a higher age-adjusted prevalence of GDM than whites, blacks, or Hispanics [7, 8, 65]. These racial/ethnic differences cannot be fully explained by differences in pre-pregnancy body mass index or obesity [66] as many Asian women with GDM do not have any historical or clinical GDM risk factors, especially excessive body weight [66]. Therefore, factors other than obesity, such genetics and lifestyle, should be explored. Asian women with TT genotype of rs7903146 polymorphism are three times as likely to have GDM as women with CC genotype.

In a genome wide association study conducted in South Korea [67] including 1,399 women with GDM and 2,025 controls, although genome-wide association between the T allele of *TCF7L2* rs7903146 polymorphism and GDM did not reach statistical significance (odd ratio of 1.499; p = 0.051), this level of association is similar to results from our overall pooled data and in Asian subgroup. In our analysis, the association between *TCF7L2* rs7903146 polymorphism and GDM in Asians is strongest under homozygous genetic model (TT vs. CC; OR 3.08; p = 0.002); however, only allele model (T vs. C allele) was used in the genome-wide association study by Kwak et al. [67].

Interestingly, we found that the at-risk T allele in Asian population varied greatly, with the most frequent percentage of 89.5% reported by a study from Malaysia [41] and the least frequent percentage of 3.3% by a study from South Korea [26], suggesting heterogeneous genetic background between Asians. It has been reported that the prevalence of GDM varies significantly among Asian and Pacific Islander subgroups, from 8.0% among Asian Indian women to 3.5% among Japanese women and 3.9% among Korean women [7]. Therefore, whenever possible, Asian and Pacific Islander subgroups should be evaluated separately in genetic health research. Interpreting genetic data from all Asian and Pacific Islanders as a group can mask important differences between many ethnic groups within this population and result in incorrect understanding of the risk for GDM in individual Asian and Pacific Islanders subgroups. The four studies included in this meta-analysis were conducted in China, South Korea, India, and Malaysia. No replicated study was conducted in the same population making more refined ethnic group analysis difficult. Future studies are needed to profile genetic risk for GDM among high risk Asian and Pacific Islander subgroups.

Result of this meta-analysis suggests the Hispanic/Latino women with TT genotype of rs7903146 polymorphism are 1.8 times more likely to have GDM than women with CC genotype. Huerta-Chagoya and associates [29] performed an association study in 750 Mexican women (408 GDM and 342 controls) and reported an association between GDM and *TCF7L2* risk haplotype (CTTC of rs7901695, rs4506565, rs7903146, rs12243326; OR 2.95, p = 2.16 x 10–06). In addition, the *TCF7L2* risk haplotype was also associated with metabolic quantitative traits, including higher levels of fasting glycemia (p = 0.0128), and 60 and 120 minutes of OGTT glycemia (p = 2.82 x 10–05, p = 0.00028, respectively). In our meta-analysis, we have included one study from Brazil [31] and two studies from Mexico [29, 32] in the Hispanic/Latino subgroup. The association between the risk T allele of rs7903146 and GDM appears to be stronger in two studies in Mexicans than the study in Euro-Brazilians, suggesting genetic differences and GDM among Hispanic/Latino subgroups require further investigation.

Characteristics of controls varied greatly between studies. For example, Cho and colleagues [26] recruited older men and women without personal or family history of T2DM as controls, however, history of GDM among non-diabetic controls were not provided. Vcelak and colleagues [49] included healthy men without family history of T2DM as controls. Even with the diverse controls, all included studies in this meta- analysis satisfied sensitivity and publication bias analyses, supporting the *TCF7L2* rs7903146 polymorphism as a risk factor for GDM.

Some limitations exist in this meta-analysis. First, we were unable to obtain detailed genotype frequency data from four published studies [43, 44, 46, 68]. Second, the statistical power of this meta- analysis is relatively small, especially for stratification analysis by ethnic subgroups. Third, our meta- analysis was to evaluate the relationship between *TCF7L2* rs7903146 polymorphism and the risk of GDM. Other factors that can affect risk of GDM, such as obesity, family history of T2DM were not included.

Fourth, geographic distribution of risk T or TT alleles of rs7903146 polymorphism can only be stratified when specific genotype data are available by study country. For example, the study by Freathy and colleagues [50] was excluded from such analysis because study subjects were from the United Kingdom and Australia, however, specific allelic frequency was not provided by country.

## Conclusion

In summary, our meta-analysis provides evidence that the T allele of the *TCF7L2* rs7903146 polymorphism is associated with GDM risk in whites, Hispanics/Latinos and Asians. Asians with homozygous TT allele of rs7903146 polymorphism have highest risk of GDM (OR = 2.08) followed by Hispanics/Latinos (OR = 1.80) and whites (OR = 1.51). The highest and lowest frequency of T allele of rs7903146 was found in Malaysia and South Korea, respectively. Future studies are needed to profile genetic risk for GDM among high risk Asian and Pacific Islander subgroups.

## Supporting Information

S1 Table PRISMA 2009 Checklist. (DOC)

S2 Table Meta-analysis on Genetic Association Studies Checklist. (DOCX)

S1 Text A List of Excluded Studies. (DOCX)
