# Supplementary material for: Transcription Factor 7-Like 2 (TCF7L2) rs7903146 Polymorphism as a Risk Factor for Gestational Diabetes Mellitus: A Meta-Analysis
Source: PLoS One. 2016 Apr 8;11(4):e0153044. doi: 10.1371/journal.pone.0153044 (PMC4825985; doi:10.1371/journal.pone.0153044)
Supplement: S1 Text — (DOCX) [file pone.0153044.s003.docx]

List of Excluded Articles

1. one article on non-human model of GDM (1)
2. two meta-analyses on association between TCF7L2 polymorphism and GDM (2, 3)
3. 13 non case-control studies (4-16)
4. 34 studies unrelated to TCF7L2 gene and GDM (17-50)
5. five studies not focus on rs7903146 polymorphism (51-55)
6. four articles lacking genotype frequency data in the text or from corresponding authors (56-59)
7. two abstracts sharing -in part- the same participants with other articles (60, 61)

1. Pasek RC, Gannon M. Advancements and challenges in generating accurate animal models of gestational diabetes mellitus. Am J Physiol-Endoc M. 2013;305(11):E1327-E38.

2. Kang S, Xie Z, Zhang D. Association of the rs7903146 polymorphism in transcription factor 7-like 2 (TCF7L2) gene with gestational diabetes mellitus: a meta-analysis. Gynecological Endocrinology: The Official Journal Of The International Society Of Gynecological Endocrinology. 2013;29(10):873-7.

3. Mao H, Li Q, Gao S. Meta-Analysis of the Relationship between Common Type 2 Diabetes Risk Gene Variants with Gestational Diabetes Mellitus. PloS one. 2012;7(9).

4. Chen PL, Yang WS. Human genetics of diabetes mellitus in Taiwan. Front Biosci. 2009;14:4535-45.

5. Klein K, Mailath-Pokorny M, Haslinger P, Knofler M, Kautzky-Willer A, Worda C. TCF7L2-polymorphism and gestational diabetes. Geburtsh Frauenheilk. 2009;69(5):447.

6. Konig M, Shuldiner AR. The genetic interface between gestational diabetes and type 2 diabetes. J Matern-Fetal Neo M. 2012;25(1):36-40.

7. Kwak SH, Jang HC, Park KS. Genetics of Gestational Diabetes Mellitus. J Korean Med Assoc. 2009;52(7):688-94.

8. Kwak SH, Kim MJ, Lee JH, Ohn JH, Kang SM, Shin HD, et al. Clinical and genetic differences between women who progress to diabetes early versus late after gestational diabetes mellitus. Diabetes. 2011;60:A347.

9. Lee SH, Demeterco C, Geron I, Abrahamsson A, Levine F, Itkin-Ansari P. Islet specific Wnt activation in human type II diabetes. Experimental diabetes research. 2008;2008:728763.

10. Petry CJ. Gestational diabetes: risk factors and recent advances in its genetics and treatment. Brit J Nutr. 2010;104(6):775-87.

11. Retnakaran R, Connelly PW, Sermer M, Zinman B, Hanley AJG. The impact of family history of diabetes on risk factors for gestational diabetes. Clinical endocrinology. 2007;67(5):754-60.

12. Robitaille J, Grant AM. The genetics of gestational diabetes mellitus: evidence for relationship with type 2 diabetes mellitus. Genet Med. 2008;10(4):240-50.

13. Watanabe RM, Allayee H, Xiang AH, Trigo E, Hartiala J, Lawrence JM, et al. Transcription factor 7-like 2 (TCF7L2) is associated with gestational diabetes mellitus and interacts with adiposity to alter insulin secretion in Mexican Americans. Diabetes. 2007;56(5):1481-5.

14. Watanabe RM, Chen Z, Richey JM, Trigo E, Lawrence JM, Xiang AH, et al. Variation in TCF7L2 is associated with longitudinal changes in acute insulin response in Mexican Americans. Diabetes. 2010;59:A342-3.

15. Wung SF, Lin PC. Shared genomics of type 2 and gestational diabetes mellitus. Annual review of nursing research. 2011;29:227-60.

16. Zhang C, Bao W, Rong Y, Yang H, Bowers K, Yeung E, et al. Genetic variants and the risk of gestational diabetes mellitus: a systematic review. Human reproduction update. 2013;19(4):376-90.

17. Anonymous. Gestational Diabetes; Investigators at Copenhagen University, Department of Obstetrics publish new data on gestational diabetes. Obesity & Diabetes Week. 2009:121.

18. Anonymous. Diabetes; New Diabetes Research from University of Southern Florida Discussed. Health & Medicine Week. 2011:791.

19. Potasso L, Perakakis N, Lamprinou A, Polyzou E, Kassanos D, Peter A, et al. Type 2 Diabetes mellitus genetic risk variant T in TCF7L2 rs 7903146 in women is associated with an increased risk for the development of gestational diabetes mellitus (GDM) with necessity of insulin therapy. Diabetologie und Stoffwechsel. 2014;9:296.

20. Bendlova B, Vcelak J, Vankova M, Lukasova P, Prazakova S, Bradnova O, et al. Association of TCF7L2 gene haplotypes with diabetes type 2, gestational diabetes but not with polycystic ovary syndrome in Czech cohorts. Diabetologia. 2009;52(S1):S139.

21. Black MH, Allayee H, Hartiala J, Xiang AH, Trigo E, Lawrence JM, et al. Interaction between transcription factor 7-like 2 (TCF7L2) and glucokinase (GCK) is associated with insulin resistance in Mexican American (MA) families of a proband with gestational diabetes (GDM). Diabetes. 2007;56:A491.

22. Cauchi S, Meyre D, Choquet H, Deghmoun S, Durand E, Gaget S, et al. TCF7L2 rs7903146 variant does not associate with smallness for gestational age in the French population. BMC medical genetics. 2007;8(1):37.

23. Caughey AB, Cheng YW, Stotland NE, Washington AE, Escobar GJ. Maternal and paternal race/ethnicity are both associated with gestational diabetes. Am J Obstet Gynecol. 2010;202(6):616.e1-.e5.

24. Cormier H, Vigneault J, Garneau V, Tchernof A, Vohl MC, Weisnagel SJ, et al. An explained variance-based genetic risk score associated with gestational diabetes antecedent and with progression to pre-diabetes and type 2 diabetes: a cohort study. Bjog-Int J Obstet Gy. 2015;122(3):411-9.

25. De Kort SWK, Mook-Kanamori DO, Jaddoe VWV, Hokken-Koelega ACS. Interactions between TCF7L2 genotype and growth hormone-induced changes in glucose homeostasis in small for gestational age children. Clinical endocrinology. 2010;72(1):47-52.

26. Ekelund M, Shaat N, Almgren P, Anderberg E, Landin-Olsson M, Lyssenko V, et al. Genetic prediction of postpartum diabetes in women with gestational diabetes mellitus. Diabetes research and clinical practice. 2012;97(3):394-8.

27. Freathy RM, Weedon MN, Bennett A, Hypponen E, Relton CL, Knight B, et al. Type 2 diabetes TCF7L2 risk genotypes alter birth weight: a study of 24,053 individuals. American journal of human genetics. 2007;80(6):1150-61.

28. Hryniewicka J, Zbucka-Kretowska M, Wawrusiewicz-Kurylonek N, Bauer W, Szamatowicz M, Telejko B, et al. Genetic variants associated with type 2 diabetes and obesity better predict gestational diabetes than traditional risk factors. Diabetologia. 2014;57(1):S448.

29. Ismail NAM, Aris NM, Mahdy ZA, Ahmad S, Naim NM, Siraj HH, et al. Single Nucleotide Polymorphism for Certain Genes Involved in Gestational Diabetes with Risk Factors and Complications Positive. Sains Malays. 2013;42(11):1613-8.

30. Kim JY, Cheong HS, Park BL, Baik SH, Park S, Lee SW, et al. Melatonin receptor 1 B polymorphisms associated with the risk of gestational diabetes mellitus. BMC medical genetics. 2011;12:82.

31. Kleinwechter H, Demandt N, Schafer-Graf U. Predisposition and phenotypes of gestational diabetes. Deut Med Wochenschr. 2014;139(21):1123-6.

32. Kuzmicki M, Telejko B, Wawrusiewicz-Kurylonek N, Kalejta K, Lemancewicz A, Zdrodowski M, et al. The expression of transcription factor 7-like 2 (TCF7L2) in fat and placental tissue from women with gestational diabetes. Diabetes research and clinical practice. 2011;94(2):e43-e6.

33. Kwak SH, Choi SH, Jung HS, Cho YM, Lim S, Cho NH, et al. Clinical and genetic risk factors for type 2 diabetes at early or late post partum after gestational diabetes mellitus. Journal of Clinical Endocrinology and Metabolism. 2013;98(4):E744-E52.

34. Liang ZX, Dong MY, Cheng Q, Chen DQ. Gestational diabetes mellitus screening based on the gene chip technique. Diabetes research and clinical practice. 2010;89(2):167-73.

35. Lyon H, Herring A, Ghosh J, Wise A, Siega-Riz AM, Stuebe A. Does maternal genotype modify the effect of exclusive breastfeeding on postpartum weight retention? Am J Obstet Gynecol. 2011;204(1):S87.

36. Mook-Kanamori DO, de Kort SWK, van Duijn CM, Uitterlinden AG, Hofman A, Moll HA, et al. Type 2 diabetes gene TCF7L2 polymorphism is not associated with fetal and postnatal growth in two birth cohort studies. BMC medical genetics. 2009;10(1):67.

37. Sayed A, Chappell S, Morgan L. The role of diabetes susceptibility genetic variants in preeclampsia. Pregnancy Hypertension. 2010;1:S71-S2.

38. Simmons RA. Role of metabolic programming in the pathogenesis of beta-cell failure in postnatal life. Rev Endocr Metab Dis. 2007;8(2):95-104.

39. Simmons RA. Developmental Origins of Adult Disease. Pediatr Clin N Am. 2009;56(3):449-66.

40. Stefanelli G, Ficarella R, Ippolito C, Manicone M, Barbaro M, Corazza C, et al. Carriers of the TCF7L2 RS7903146 variant with GDM have an increased risk of developing impaired glucose regulation after pregnancy. Diabetes. 2012;61:A342.

41. Stuebe A, Lyon H, Herring A, Wise A, Ghosh J, Siega-Riz AM. Diabetes and obesity risk allele carriage, pregravid BMI and gestational weight gain. Am J Obstet Gynecol. 2009;201(6):S219.

42. Stuebe AM, Wise A, Nguyen T, Herring A, North KE, Siega-Riz AM. Maternal genotype and gestational diabetes. American journal of perinatology. 2014;31(1):69-76.

43. Wang Y, Nie M, Li W, Ping F, Hu YY, Ma LK, et al. Association of Six Single Nucleotide Polymorphisms with Gestational Diabetes Mellitus in a Chinese Population. PloS one. 2011;6(11):e26953.

44. Wawrusiewicz-Kurylonek N, Kuzmicki M, Szamatowicz J, Gorska M, Kretowski AJ. The TCF7L2 expression in subcutaneous and visceral fat tissues in women with gestational diabetes. Diabetologia. 2008;51:S336-S.

45. Yaghootkar H, Freathy RM. Genetic origins of low birth weight. Curr Opin Clin Nutr. 2012;15(3):258-64.

46. Zhang Y, Xiao X, Zhang Z, Ma X, Xu T, Li W, et al. Role of high-risk variants in the development of impaired glucose metabolism was modified by birth weight in Han Chinese. Diabetes / Metabolism Research And Reviews. 2015;31(8):790-5.

47. Zhang ZW, Liu CY, Yu N, Guo W. Removable uterine compression sutures for postpartum haemorrhage. Bjog-Int J Obstet Gy. 2015;122(3):429-33.

48. 周琦, 李伟, 郑景晨, 聂敏, 孙梅励, 张葵, et al. KCNQ1基因单核苷酸多态性与妊娠期糖尿病相关性研究. 生殖医学杂志. 2010;1:40-7.

49. 胡晓菡, 郑洁, 张葵. 江苏地区2型糖尿病易感基因单核苷酸多态性与妊娠期糖尿病关系的研究. 国际检验医学杂志. 2014;10:1245-7.

50. Potasso L, Perakakis N, Lamprinou A, Polyzou E, Kassanos D, Peter A, et al. The type 2 diabetes genetic risk variant TCF7L2 rs7903146 is differentially associated with gestational diabetes: differences between central and Mediterranean Europeans. Diabetologia. 2012;55:S443-4.

51. Pagan A, Sabater M, Olza J, Prieto-Sanchez MT, Blanco-Carnero JE, Parrilla JJ, et al. A gene variant in the transcription factor 7-like 2 (TCF7L2) is associated with an increased risk of gestational diabetes mellitus. Ann Nutr Metab. 2013;63(suppl 1):1319.

52. Lopez-Reyes R, Malacara JM, Perez-Luque EL. Association of rs7901695 and rs12255372 of TCF7L2 gene with gestational diabetes mellitus and its relationship with metabolic and hormonal characteristics in Mexican women. The Endocrine Society's 95th Annual Meeting and Expo; June 15-18, 2013; San Francisco.

53. Stuebe A, Lyon H, Herring A, Wise A, Joyee G, Siega-Riz AM. Diabetes risk allele carriage, pregravid BMI and risk of gestational diabetes. Am J Obstet Gynecol. 2009;201(6):S102.

54. 王庆玲, 王义, 郭健, 张树荣, 张静. TCF7L2基因rs290487位点多态性与妊娠期糖尿病发病相关性的探讨. 中国妇幼保健. 2013;18:4374-5.

55. 回园敕, 平凡, 李伟, 聂敏, 张丽红, 黎明, et al. TCF7L2基因单核苷酸多态性与妊娠期糖尿病的相关性研究. 中华内分泌代谢杂志. 2011;1:32-5.

56. Huopio H, Cederberg H, Vangipurapu J, Hakkarainen H, Pääkkönen M, Kuulasmaa T, et al. Association of risk variants for type 2 diabetes and hyperglycemia with gestational diabetes. European journal of endocrinology / European Federation of Endocrine Societies. 2013;169(3):291-7.

57. Katsarou A, Lynch K, Shaat N, Hakansson R, Nilsson A, Lernmark B, et al. Gestational diabetes is associated with a common variant of the transcription factor 7 like 2 (TCF7L2) gene. Diabetologia. 2007;50:S138.

58. Khder A, Al-Khinji M, Saleh R, Rooshenas AA, Rizk NM. Association between carriers of TCF7L2 polymorphisms, insulin and glucagon-like peptide-1 among Arab pregnant women. The Endocrine Society's 95th Annual Meeting and Expo; June 15-18, 2013; San Francisco.

59. Potasso L, Perakakis N, Lamprinou A, Polyzou E, Kassanos D, Peter A, et al. Type 2 Diabetes mellitus genetic risk variant T in TCF7L2 rs 7903146 in women is associated with an increased risk for the development of gestational diabetes mellitus (GDM) with necessity of insulin therapy. Diabetologie und Stoffwechsel. 2014;9.

60. Vejrazkova D, Lukasova P, Vankova M, Bradnova O, Halkova T, Vcelak J, et al. Genetic background of gestational diabetes mellitus in the Czech population. Diabetologia. 2013;56:S511.

61. Potasso L, Perakakis N, Lamprinou A, Polyzou E, Kassanos D, Peter A, et al. Type 2 Diabetes mellitus genetic risk variant T in TCF7L2 rs7903146 in women is associated with an increased probability of insulin therapy in gestational diabetes mellitus (GDM). Exp Clin Endocr Diab. 2014;122(3):P096.
